# Research on the Development of Hospital Intelligent Finance Based on Artificial Intelligence

**DOI:** 10.1155/2022/6549766

**Published:** 2022-08-09

**Authors:** Mengxuan Ma

**Affiliations:** Hubei Cancer Hospital, Tongji Medical College, Huazhong University of Science and Technology, Wuhan, Hubei 430079, China

## Abstract

Based on the development background of the interweaving and integration of computer technology and Internet technology, China's artificial intelligence industry is quietly rising. In the social life of the information age, the artificial intelligence industry represented by machine deep learning is playing a very important role. This study in combination with the background of the new health reform, in view of the reform of the medical industry, analyzes the connotation of financial wisdom based on the important role of the hospital financial development problems, puts forward the development direction of artificial intelligence hospital financial wisdom development measures, designed to meet the changing external environment demand, reduces human costs, and improves the overall efficiency of hospital financial fund management. Based on the evaluation results, this paper proposes the correct direction for the development of hospital intelligence finance by using the BP neural network model. After the analysis, it is found that the development of artificial intelligence is an important measure to promote the development of hospital intelligent finance. In other words, hospital intelligent financial management is the product of the continuous progress of artificial intelligence technology. At the present stage, the intelligent financial management problems of hospitals are mainly as follows: (1) lack of financial informatization, (2) lack of perfect financial risk early warning system, and (3) the phenomenon of “information island” in the financial system. After analyzing the above problems, the research believes that the development of hospital intelligent finance based on artificial intelligence needs to solve the above thorny problems, so as to improve the outcome of hospital intelligent finance development. The following work should be done: (1) strengthen the design of information sharing module, (2) intelligent control of the cost of hospitals, and (3) intelligent treatment of hospital accounting. Combining the development of artificial intelligence and hospital intelligent finance theory, combined with the actual trend of financial intelligence development under the background of artificial intelligence development in the new era, it provides scientific basis for the development of hospital intelligent finance.

## 1. Introduction

In a broad sense, financial management includes accounting, tax, audit, budget, and debt management, as well as investment, financing, and operation decisions [1–4]. China's financial management is divided into the following five development stages: (1) traditional stage, (2) computerization stage, (3) information stage, (4) intelligent stage, and (5) smart stage. “Smart finance” management is the product of the continuous progress of artificial intelligence technology, which provides a more novel path and a broader space for the transformation of the financial management field [5]. Its essential feature lies in the more scientific and humanized management of value flow in economic activities, which is reflected in the organic combination of actual business development of hospitals, universities, and other entity organizations, which can liberate human resources and improve the ability to analyze and judge the judgment of financial status [6]. Starting from the development process of artificial intelligence and the superiority of intelligent finance, this paper discusses the innovative scheme of intelligent finance.

## 2. Problem Analysis

### 2.1. Insufficient Degree of Financial Informatization

**Figure 1:**
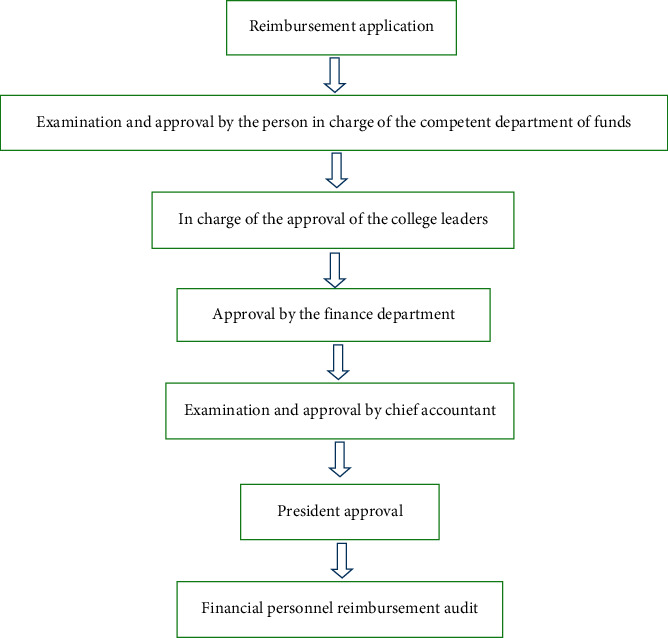
Traditional financial reimbursement process.

At present, the degree of financial informatization in most hospitals is not high, and the financial informatization is mainly reflected in accounting, but other financial work is still manually recorded, and the work efficiency is not high [7]. For example, many hospitals in the budgeting and budget implementation or through the financial personnel are using Excel forms for manual registration and summary [8]. As the general hospital involves multiple departments, with the continuous expansion of economic scale, there are more and more economic problems. In the process of manual registration and summary, it is easy to miss a recording, which will affect the budget preparation and implementation results [9]. For another example, in many hospitals in the financial reimbursement process, reimbursement application filling, form approval, reimbursement original attachments, and access to accounting vouchers and other links are manual. The degree of information is not high [10]. Traditional financial reimbursement process is generally divided into seven steps. The details are shown in Figure 1.

In the above mode, there may be multilevel approval according to the business needs, and the reimbursement agent needs to find different approval personnel for reimbursement approval [11]. Because the examination and approval personnel are mostly administrative leaders, the official duties are heavy, which may cause the phenomenon of the reimbursement agents running empty and reduce the reimbursement efficiency [12]. For the examination and approval personnel, the daily scattered and multiple examination and approval signatures take up a lot of time, and each examination and approval signature will also interrupt the working ideas of the examination and approval personnel, affecting the work efficiency. Financial personnel manually calculate the reimbursement amount and prepare accounting vouchers [13]. With the increasing volume of hospital business, there are more and more economic matters, and the efficiency of manual reimbursement is not high and easy to make mistakes[14]. If “mobile approval” and “online reimbursement” can be realized through information technology, the reimbursement operator can be reduced and the efficiency of financial reimbursement can be improved.

### 2.2. Lack of Perfect Financial Risk Early Warning System

Under the traditional financial mode of hospitals, postmanagement is the main management method of financial risks [15]. For financial management risks, there is a lack of prewarning and in-process control and a lack of dynamic financial risk management mechanism, which makes the financial risk management in hospital financial management have an obvious lag. In the financial management of hospitals, cash flow risk is the main financial risk faced [16]. For example, under the COVID-19 outbreak, some hospitals have closed all or some outpatients, resulting in a sharp decline in outpatients and chronic inpatients, the number of outpatient visits, and inpatients, and a sharp decline in medical income and cash flow. At the same time, the current cost of medical protective equipment, disinfection, and other equipment increased significantly, resulting in a significant increase in cash flow expenditure [17]. The decrease of cash inflows and the increase of cash outflow make the hospital face huge financial problems, and the capital situation is severe [18]. Therefore, hospitals should strengthen the control of financial risks, especially cash flow, and establish a dynamic financial risk early warning system covering the whole process of economic business.

### 2.3. “Information Island” Problem

The financial system of most hospitals is completely connected with hospital information system, assets, and personnel management, and cannot realize information sharing. The problem of “information island” is very serious [19]. At the same time, because different suppliers provide different information systems, and the standards and specifications between each system are different, compatibility cannot be achieved, resulting in the effective integration of data resources between systems. For example, outpatient charges, hospitalization charges, and other income information. In the hospital information system, it is difficult to timely connect and share with the financial accounting system in real time. Therefore, the daily medical income and other accounting is still in the state of manual accounting [20]. The lack of connection between finance and purchase and supply system easily leads to the hospital material flow, capital flow, and information flow that cannot effectively form a virtuous cycle, which may cause the failure to match the accounts and the lack of data authenticity. At the same time, if the information is not effectively managed, it is difficult to carry out accurate financial accounting, and the operation and management problems cannot be found in time. It causes a lot of data collection or collation duplication, wastes a lot of resources, and seriously affects the efficiency of financial management.

## 3. The BP Neural Network Model

### 3.1. Algorithm of BP Neural Network Model under Hospital Intelligent Financial Model

#### 3.1.1. Part 1: Basic Structure

**Figure 2:**
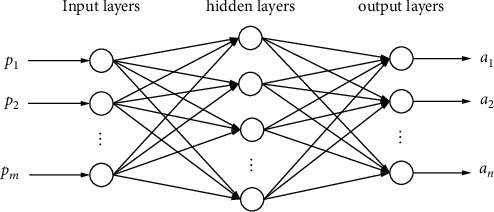
Basic structure.

**Figure 3:**
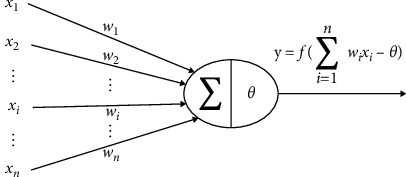
Structure of each neuron model.

Common neural networks can be divided into three parts: input layer, output layer, and several hidden layers.  Figure 2 is a typical three-layer neural network structure and Figure 3 is the structure of each neuron model.

‘*X*_*n*_' represents the input value of the ‘*n*' neuron, ‘*W*_*n*_' represents the connection weight value of the ‘*i*'neuron, ‘*θ*' is threshold values, ‘*Y*_*i*_' is the output value of the ‘*i*' neuron. The resulting activation function is as follows:

(1) $$\begin{array}{c} y=f\left(\sum_{i=1}^{n}w_{i}x_{i}-\theta \right), \end{array}$$

where the activation function refers to the introduction of nonlinear factors in the neurons, so that the neural network can be arbitrarily close to any nonlinear function. Sigmoid function, tan*h* function, and ReLU function are relatively extensive activation functions used in academic sessions. The threshold is a limited value. The difference result after the sum of ‘*W*_*i*_*X*_*i*_' is finally expressed as inhibitory or activation events and gives the output result, which generally adopts the binary scientific counting method. If the difference is less than or equal to 0 and *Y* = 0, state indicates inhibition. If the difference is greater than 0 and *Y* = 1, state indicates activation.

#### 3.1.2. Part 2: BP Training Method

To train a BP neural network, it is the adjustment and optimization of two parameters: weight and bias. The premise that the neural network can accurately reflect the training results is that the model is fully trained, and the optimal model parameters are obtained through the training: the connection weight ‘*W*' and ‘*θ*'. The main training method of parameter learning is the BP algorithm, which is mainly based on the gradient descent algorithm, and it is also a common algorithm used to train the neural network model parameters in practical work. The main working principle of the gradient descent method is to solve the optimal solution of the parameters along the direction of the fastest local descent.

Two processes of the BP algorithm are forward and back propagation. The forward propagation is the signal, and the reverse propagation is the error. The error values are backpropagated without a given error range to correct the cell weights. Since the learning of parameters in the BP algorithm is based on the gradient descent algorithm, the core of gradient descent is the calculation of gradient. The training of neural networks is generally divided into four processes. The first process travels forward to calculate the error value between the output value and the actual value. The second process backpropagation calculates the contribution value of all neurons to the total error in each layer, mainly two classes of values of the output and hidden layers. A third process gradient was calculated to find the gradient of the total error against each model parameter for parameter weights and threshold updates. The fourth process parameter is updated, updating the weights and thresholds.

#### 3.1.3. Part 3: Select the Parameter

The number of nodes in the input layer is the number of input neurons. The number of points in the output layer is divided according to the analysis. If it is a classification problem, the corresponding number of nodes is the number of classification. If it is a regression problem, the corresponding number of nodes is equal to 1. The number of layers and the number of nodes in the hidden layer will make the model too pleasing to the training set and prone to overfitting, while the number of hidden layers and the number of hidden layer nodes are too small, underfitting will occur. There is no way to accurately determine the number of hidden nodes, and the specific number of hidden nodes can be formulated according to the empirical formula.

(2) $$\begin{array}{c} l<n,l<\sqrt{n+k}+i,l<2\sqrt{n}. \end{array}$$

In the abovementioned formula, ‘*l*' represents the number of nodes in the hidden layer, ‘*n*' represents the number of nodes in the input layer, ‘*k*' represents the number of nodes in the output layer, and ‘*i*' represents any constant between 0 and 9.

#### 3.1.4. Part 4: Data Were Normalized

Data normalization is very important. The input layer will involve a variety of different indicators. These indicator dimensions and dimensional units are different, and different indicators will directly feedback the inaccuracy of the data results. The purpose of data standardization processing is to eliminate the influence of dimension, make the data index in the same order of magnitude, and each index is suitable for comprehensive comparative evaluation. The method used in this study is the normalization method, which changes the number into a decimal between 0 and 1. We get the following formula:

(3) $$\begin{array}{c} X_{n}\left(\text{new}\right)=\frac{X_{n}-X_{\min}}{X_{\max}-X_{\min}}. \end{array}$$

In the abovementioned formula, ‘*X*_*n*_' represents the original indicator, ‘*X*_max_' represents the maximum of ‘*X*' in all data, ‘*X*_min_' represents the minimum of ‘*X*' in all data, and ‘*X*_*n*_ (new)' is the new value obtained by normalization the original index ‘*X*_*n*_'.

### 3.2. BP Neural Network Model Data Processing

This study mainly uses MATLAB programming software to train and simulate the results of neural networks. The data mainly comes from the background financial data randomly selected from the intelligent finance development research of a hospital. The initial data sample has 3790 data sets, and the data classification is shown in Table 1.

In this study, the data were imported into MATLAB software for analysis. Based on the normalization method, all 3790 sets of data were the decimal between (0,1). After the normalization analysis, 3000 sets of data were randomly selected as the training set, and the remaining 581 sets of data were used as the experimental set. On this basis, the accuracy of the model prediction is verified. The training step was 500, the expected error target was 0.001, and the learning step was 0.01.

In the above experiments, the number of hidden layer nodes was set to 7, 8, 9, 10, and 11, and the BP neural network model was trained by using the training set data. This paper mainly examines the training effect of different hidden nodes, from the following two aspects: first, to analyze the overall number of credit level identification errors. Second, to analyze the relative error of the deviation degree alone.

(4) $$\begin{array}{c} \text{error}=\text{Average}\left[\frac{\text{abs}\left(\text{Model output value}-\text{experimental value}\right)}{\text{experimental value}}\right]. \end{array}$$

For the regression prediction model, if the prediction can draw the predicted value and the real value in the same coordinate system. In the actual model, the judgment coefficient *R*^2 is usually used to evaluate the actual results of the regression model, which is to evaluate the degree to which the regression model explains the changes in the dependent variable *y*. The *R*^2 values range from 0–1, a percentage is usually used for the representation. If a regression model has an *R*^2 = 0.7, then this regression model is 70% interpretable of the predicted results. The academic circle agrees that *R*^2 is greater than 0.75, with a better model fit, and a high degree of interpretability. If R^2 is less than 0.5, it can be considered problematic in model fitting and not suitable in regression analysis.

The *R*^2 regression model was evaluated using the MATLAB model. The *R*^2 = 0.99351 calculated from the scoring data of the experimental set also reflects the good ability to estimate the financial risk in this paper and the high accuracy from the side. If this model is applied to the actual process of scoring financial indicators, it can help the hospital to accurately determine the score of each financial risk, so as to effectively help the hospital make a reasonable judgment of the estimated financial risk situation.

This chapter is mainly the simulation of the financial intelligence of the hospital neural network model, according to multiple secondary indicators. Through MATLAB software, using BP neural network model for model training and simulation and through the good simulation results of the model, it proved that the model can predict and evaluate the development of financial intelligence and can effectively help hospitals to predict financial risks, so as to reduce the possible fund management risk. In the future, the training effect can be improved by enriching and optimizing evaluation indicators, increasing training models, improving algorithms and other methods, and enhancing the interpretation ability of the neural network, so that the model can more accurately predict financial management risks, reduce bad debts, and improve the financial intelligence level of the hospital.

## 4. Effective Measures of Intelligent Financial Mode of Hospitals under Artificial Intelligence

### 4.1. Strengthen the Information Sharing Module Design

Information sharing is mainly aimed at the efficient exchange of financial data in hospitals. In this mode, the network information platform can be used to integrate the financial data information of independent accounting units of each department, push the necessary financial data to the financial management personnel, and then define the content, and corresponding treatment plans are set up for classified control. Due to the different specific functions of hospital departments, data interaction and information transmission should be carried out according to the actual work and the principles of orderly, reasonable, and complete work so as to avoid repeated transmission and transmission of wrong information. In the specific preparation, the appropriate report template is selected in the system database according to the business development, and then the report data is retrieved according to the requirements of the template. The platform can screen, integrate, and summarize the data, and write the analysis and explanation of the report. Finally, realize the information exchange of independent financial subjects through the interactive platform. At the same time, the financial personnel can adjust the format of the financial report from the needs of themselves and the unit, carry out a comprehensive analysis and interpretation, focus on the surface abnormal data, and submit it to the management for review.

### 4.2. Intelligent Control of Hospital Costs

An important function of intelligent finance is accounting treatment, namely accounting function. The intelligent construction of the reimbursement module is to realize the automation and intelligent operation of a series of work such as voucher recording, storage, and bookkeeping in the interactive system. Due to the introduction of AI technology, the system can automatically identify the image content and use big data analysis processing. With the popularity of electronic invoice, intelligent financial system, invoice system depth interaction, and implement business credentials recorded in the core accounting system, the paper invoice content through AI identify key data such as billing, tax price, tax into digital language stored in the cloud. At the same time, intelligent finance can connect with the tax invoice query system to identify the authenticity of invoices and effectively control false businesses.

The financial personnel of the hospital shall submit the paperless examination and approval application in the OA office system after the system examination according to the established authority setting, and the process can be signed and approved in the OA system. After the OA review, the original voucher approval form and approval details will be pushed to the financial accounting system. The financial personnel will review, and the document information will be encrypted by the smart financial system and will be displayed in the form of QR code on the documents, so as to realize the uniqueness and confidentiality of the data. Subsequently, the system automatically prints out the paper version of the accounting reimbursement documents, and the financial personnel will put the documents and the original vouchers into the accounting file cabinet in accordance with the provisions. The service mode of “smart finance” has the ability of memory feedback, which can analyze the daily entry writing rules of financial personnel and automatically make accounting entries for the identified economic business data, saving time and facilitating the follow-up review. At the same time, the data before and after the review can coexist, and it can be accurately and comprehensively be classified and archived according to the requirements of accounting standards. In the financial budget processing, “smart finance” service can handle the accounting vouchers according to the procedures. To analyze the financial operation data of the previous 3–5 years, a variety of budget models is combined to make a preliminary calculation of the capital flow of the future economic business. Then, the key indicators of the current market environment and policy situation changes is inputted into the system based on various factors to make a reasonable judgment on the cash inflow and outflow of the unit, capital demand, business prospects, and asset status. On this basis, the financial budget is drafted. After a review of the budget unit based on the opinions and suggestions of the budget subject, the next module is transferred for execution.

### 4.3. Intelligent Treatment of Hospital Accounting

Cost management accounting is one of the key links in the financial management, “financial” system has intelligent cost accounting and management module, and it confirms the current profit and loss accounting cost and cost responsibility center in time after the economic business occurs according to the cost accounting rules of the cost entity. If it cannot be confirmed, it can predict according to the past practice and conduct liquidation after the end of the accounting cycle. The cost management module mainly ensures that the accounting items can cover all links of the unit's economic business. In cost control, it divides and counts the costs of independent economic departments, and analyzes the actual economic benefits of independent financial units through cost analysis models, structural analysis, and trend prediction methods. The cost control intelligence module can prepare analysis reports for the management to use when adjusting the organizational management structure and business form.

## 5. Conclusion

The “smart” financial service model in the era of artificial intelligence has played a profound and revolutionary role in the reform of financial operation mechanism. It can strengthen the integration of modern intelligent technology and management methods, strengthen the training of financial business talents, make them adapt to the financial management mechanism under the background of artificial intelligence, and deepen the “smart” service mode to better play the positive role of financial management.

## Figures and Tables

**Table 1:** Data classification.

Credit rating	*A*	*B*	*C*	*D*	*E*	*F*	Total
Sample size	41	1314	1864	428	129	14	3790

## Data Availability

Data are available upon request from the corresponding author.

## References

[1] Sui L. G., Chen J. Y. (2013). Based on the Internet of things and cloud computing technologies to develop smart finance. *Journal of Changchun Finance College*.

[2] Li L., Lei B., Mao C. (2022). Digital twin in smart manufacturing. *Journal of Industrial Information Integration*.

[3] Routledge (2014). Infrastructure planning and finance: a smart and Sustainable Guide for Local Practitioners. *Reprints and Permissions*.

[4] Li L., Qu T., Liu Y. (2020). Sustainability assessment of intelligent manufacturing supported by digital twin. *IEEE Access*.

[5] Hu N. . (2011). Discussion of shanghai smart finance development. *Journal of Shanghai Fisheries University*.

[6] Fan Ea -Ivanovici M., Pan M. C. (2022). Crowdfunding as a smart finance and management tool: institutional determinants and well-being considerations. *Evidence from Four Central and Eastern European Countries*.

[7] Focus I. C. (2011). IIASA. Carbon market and climate finance for climate-smart agriculture in developing countries. *Climate Focus Inc*.

[8] Hu N., Wang Y. Analysis of building cloud-based smart finance platform of Shanghai.

[9] Lang L. E. (2011). Financial points of entry. Sovereign wealth funds: hidden security threat or smart finance?. *Georgetown University*.

[10] Hayes T. (2011). Smart finance: claiming GST paid on water termination fees. *Irrigation Australia the Official Journal of Irrigation Australia*.

[11] Huston S., Rahimzad R., Parsa A. (2015). *SmartSURFinance HustonReyhanehParsa 23April2014*.

[12] Huston S., Rahimzad R., Parsa A. (2014). Smart’ finance for sustainable urban regeneration. *Social Science Electronic Publishing*.

[13] Setyadi D., Soegiarto H. E., Latif I. N. (2016). Sistem dan prosedur penjualan kredit sepeda motor pada Pt. *Smart Multi Finance Kecamatan Melak Kabupaten Kutai Barat*.

[14] Glossbrenner A. (2011). Smart guide to managing personal finance. *John Wiley & Sons*.

[15] Shofner S. L. (2000). The impacts of smart growth on municipal finance: perspectives of city planning directors. *Across Texas*.

[16] Jas Per K., Sung H. K. (2012). CSO-state partnerships and social finance: smart social capital and shared incentives towards public-private partnership efficiency using social impact bonds. *Social Science Electronic Publishing*.

[17] Lang R. E., Lefurgy J., Hornburg S. (2010). From wall street to your street: new solutions for. *Smart Growth Finance*.

[18] Peng J. The study of the development and application situation of smart finance.

[19] Guo P. (2019). Linqu county build fiscal big data application platform and contribute to the construction of “smart finance”. *Fiscal Science*.

[20] Witthaut M., Deeken H., Sprenger P. (2017). Smart objects and smart finance for supply chain management. *Logistics Journal nicht-referierte Veröffentlichungen*.

